# Suppressing Nitrite-oxidizing Bacteria Growth to Achieve Nitrogen Removal from Domestic Wastewater via Anammox Using Intermittent Aeration with Low Dissolved Oxygen

**DOI:** 10.1038/srep13048

**Published:** 2015-09-10

**Authors:** Bin Ma, Peng Bao, Yan Wei, Guibing Zhu, Zhiguo Yuan, Yongzhen Peng

**Affiliations:** 1 Key Laboratory of Beijing Water Quality Science and Water Environment Recovery Engineering, Beijing University of Technology, Beijing, China; 2 State Key Laboratory of Environmental Aquatic Quality, Research Center for Eco-Environmental Sciences, Chinese Academy of Sciences, Beijing, China; 3 Advanced Water Management Center, The University of Queensland, St Lucia, QLD 4072, Australia

## Abstract

Achieving nitrogen removal from domestic wastewater using anaerobic ammonium oxidation (anammox) has the potential to make wastewater treatment energy-neutral or even energy-positive. The challenge is to suppress the growth of nitrite-oxidizing bacteria (NOB). This study presents a promising method based on intermittent aeration with low dissolved oxygen to limit NOB growth, thereby providing an advantage to anammox bacteria to form a partnership with the ammonium-oxidizing bacteria (AOB). The results showed that NOB was successfully suppressed using that method, with the relative abundance of NOB maintained between 2.0–2.6%, based on Fluorescent *in-situ* Hybridization. Nitrogen could be effectively removed from domestic wastewater with anammox at a temperature above 20 °C, with an effluent total nitrogen (TN) concentration of 6.6 ± 2.7 mg/L, while the influent TN and soluble chemical oxygen demand were 62.6 ± 3.1 mg/L and 88.0 ± 8.1 mg/L, respectively.

Nitrogen must be removed from wastewater to protect rivers and other water bodies from eutrophication. In conventional wastewater treatment plants (WWTPs), aerobic nitrification and anoxic denitrification are widely used to remove nitrogen. A lot of energy is required for aeration to create aerobic conditions for nitrification. Meanwhile, organic matter is needed for denitrification. With the discovery of anaerobic ammonium oxidation (anammox)^1^, autotrophic nitrogen removal can be achieved without the need of organic carbon, because anammox bacteria can reduce nitrite with ammonium as the electron donor and with CO_2_ as the carbon source for growth^2^. As such, organic matter in wastewater is not needed for nitrogen removal, and can instead be anaerobically converted to biogas (CH_4_) for maximum energy recovery from wastewater. At the same time, energy consumption can also be reduced because oxygen demand is reduced by 60% in a biological nitrogen removal system using anammox^3^. As such, anammox has been recognized as an attractive alternative nitrogen removal process^4^. This technology has been successfully applied to treat wastewater containing a high-level of ammonium, such as sludge reject water. However, the nitrogen contained in this stream represents only approximately 25% of the total nitrogen (TN) load in a WWTP^5^. If the anammox process could also be applied to remove the nitrogen contained in raw sewage, WWTPs could become energy-neutral or even energy-positive^4^.

In a biological nitrogen removal process with anammox, part of the ammonium in wastewater is oxidized to nitrite by ammonium-oxidizing bacteria (AOB). Then, anammox bacteria oxidize the remaining ammonium to nitrogen gas using nitrite produced as the electron acceptor (Fig. 1). So, the premise of achieving anammox is the enrichment of AOB and anammox bacteria in the system. The retention of AOB is not a problem since it is widely present in conventional biological nitrogen removal wastewater treatment systems. The doubling time for anammox bacteria and pure AOB is 5.5–7.5 day^6^ and 7–8 h^7^, respectively. Therefore, retaining anammox bacteria is considered to be a bottleneck for achieving the anammox process in sewage treatment systems, especially at low temperatures. Recently, good retention of anammox bacteria was achieved by forming granular sludge in an upflow anaerobic sludge blanket reactor treating sewage^8^, and these granules could work well at 12–30 °C^8^,^9^. This indicates that, with the use of granular sludge, the retention of anammox bacteria in sewage treatment is no longer a problem.

Suppressing the growth of nitrite-oxidizing bacteria (NOB) is another important factor for achieving nitrogen removal from sewage via the anammox reaction. If NOB, which oxidizes nitrite to nitrate under aerobic conditions, coexists with anammox bacteria in the system, nitrite will be rapidly consumed by NOB. Consequently, the anammox bacteria growth will be limited due to an inadequate supply of nitrite. However, studies suggest that controlling NOB growth is difficult in a simultaneous nitritation and anammox reactor treating low strength wastewater^10^,^11^. Thus, suppressing NOB growth is a real bottleneck for achieving autotrophic nitrogen removal via anammox in sewage treatment systems^12^.

Nitrite, a substrate for nitrite oxidation carried out by NOB, is the product of ammonium oxidation. As such, nitrite oxidation always lags behind ammonium oxidation. Consequently, if aeration is turned off when nitrite starts accumulating in the reactor, the accumulated nitrite would be consumed by anammox bacteria (Fig. 1), resulting in less NOB growth compared to the case with constant aeration. The application of such conditions over time would lead to the gradual reduction in the NOB population.

Based on the above analysis, we hypothesize that intermittent aeration with low dissolved oxygen (DO) may be a promising method for suppressing NOB growth in a one-stage nitritation/anammox reactor for treating domestic wastewater. Intermittent aeration with low DO results in an alternating aerobic (low DO) and anoxic operating condition. Under low DO aeration, nitrite easily accumulates, because the apparent growth rates of AOB (μ_AOB_) are faster than that of NOB (μ_NOB_) under low DO^13^. Once nitrite accumulates, aeration is switched off and the reactor enters into an anoxic phase, enabling nitrite consumption by anammox bacteria. When the anammox reaction finishes, the next aeration stage begins. Under these operating conditions, the NOB growth is likely less than that of AOB in the aeration stage. At the same time, anammox bacteria could have a significant advantage over NOB for nitrite.

This study investigated the feasibility of using intermittent aeration with low DO to suppress NOB growth for achieving nitrogen removal from domestic wastewater via anammox. There were three tasks. First, NOB growth was studied by measuring the abundance of NOB in activated sludge after applying intermittent aeration with low DO. Meanwhile, the AOB and anammox bacteria abundance was also investigated, to understand the dynamics of these key organisms. Second, the role of intermittent aeration with low DO in suppressing NOB growth was studied, by investigating the nitrogen conversion pathways in the reactor. Finally, the nitrogen removal performance was studied.

## Results

### Microbial community structure changes after applying intermittent aeration with low DO

**Figure 2:**
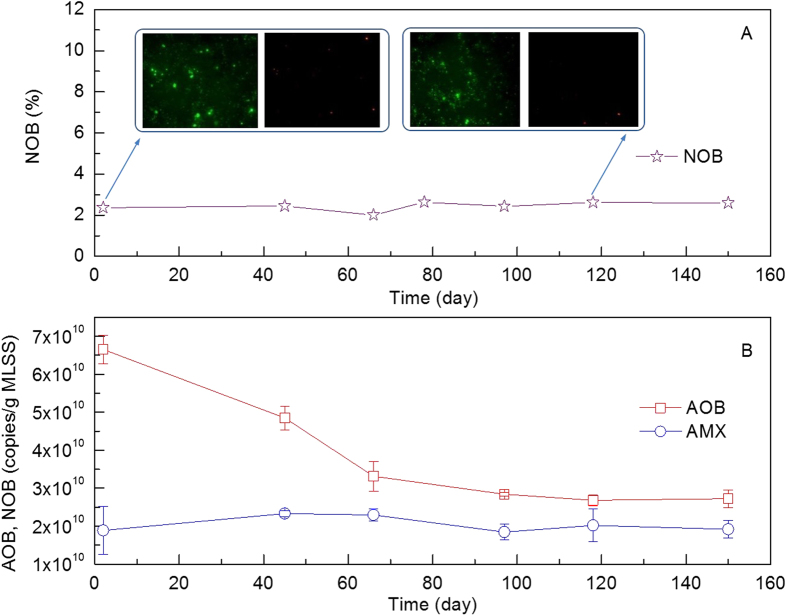
Relative abundance of NOB in the activated sludge (**A**) and the copy numbers of AOB and anammox bacteria (**B**) per gram of the dry activated sludge. Eubacteria are shown in green and NOB are shown in red (**A**).

Suppression of NOB was observed after applying intermittent aeration with low DO (Fig. 2). The relative abundance of NOB was 2.0–2.6% throughout the operational time. The seeding sludge, which was obtained from a pilot-scale nitritation/anammox reactor treating N-rich wastewater, consisted of NOB with a relative abundance of 2.4%. After 150 days operation, NOB remained at 2.6%.

Meanwhile, the AOB and anammox bacteria were maintained in the reactor. The abundance of anammox and AOB bacteria were detected with qPCR based on the specific anammox hzsB functional gene. The copy number of AOB was initially 6.66 ± 0.38 × 10^10^ copies/g MLSS; it decreased gradually and stabilized at 2.69 ± 0.14 × 10^10^ copies/g MLSS on day 118 (Fig. 2). The abundance of anammox bacteria was maintained between 1.89 ± 0.62 × 10^10^ copies/g MLSS and 2.33 ± 0.08 × 10^10^ copies/g MLSS.

**Figure 3:**
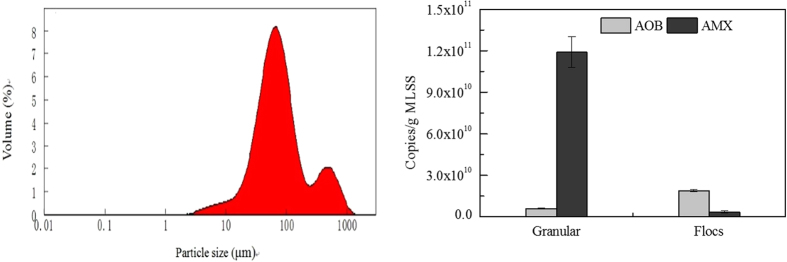
Activated sludge size distribution, copy numbers of ammonium oxidizing bacteria (AOB) and anaerobic ammonium oxidation bacteria (AMX) per gram of the dry granular sludge (>200 μm) and activated sludge flocs (<200 μm).

The AOB and anammox bacteria distribution in the two forms of sludge was further investigated on day 70, when TN removal efficiency was at a relatively stable level, with an average efficiency of 85%. The particle size distribution curve shows two clear peaks at 70 μm and 530 μm, respectively (Fig. 3A). These peaks likely corresponded to the mean particle sizes of flocs and granules. Based on this result, the particles were separated using a wet-sieving method with a seive of 200 μm (delineating the valley between two peaks in Fig. 3A). Particles larger than 200 μm were broadly regarded as granules, with a mean size of 522 um; particles smaller than 200 μm were characterized as flocs, with a mean size of 67 μm (Figure S1). The settling velocity of these granules (>200 μm) fell in the range of 17–60 m/h, consistent with the settling velocity of anammox granular sludge (Table S1).

Then, the abundance of AOB and anammox bacteria were investigated in the granules and flocs (Fig. 3B). The granular sludge mainly contained anammox bacteria with a copy number of 1.19 ± 0.11 × 10^11^ copies/g MLSS. While it still contained AOB, the abundance was much lower, with a copy number of 5.94 ± 0.39 × 10^9^ copies/g MLSS. In contrast, sludge flocs mainly contained AOB, with an abundance of 1.88 ± 0.08 × 10^10^ copies/g MLSS, in comparison with a copy number of anammox bacteria at 3.54 ± 0.63 × 10^9^ copies/g MLSS.

This result is in good agreement with several previous studies, which showed that anammox grew in bigger granules, while NOB grew in smaller ones^14^,^15^,^16^. Hubaux *et al.*^17^ reported that the optimal DO concentration of the nitritation/anammox granular sludge reactor with flocs is lower than in the reactor without flocs. This means that the energy consumption of the nitritation/anammox granular sludge reactor with flocs could be lower, since aeration cost could be reduced.

### Nitrogen conversion in the nitritation/anammox reactor

**Figure 4:**
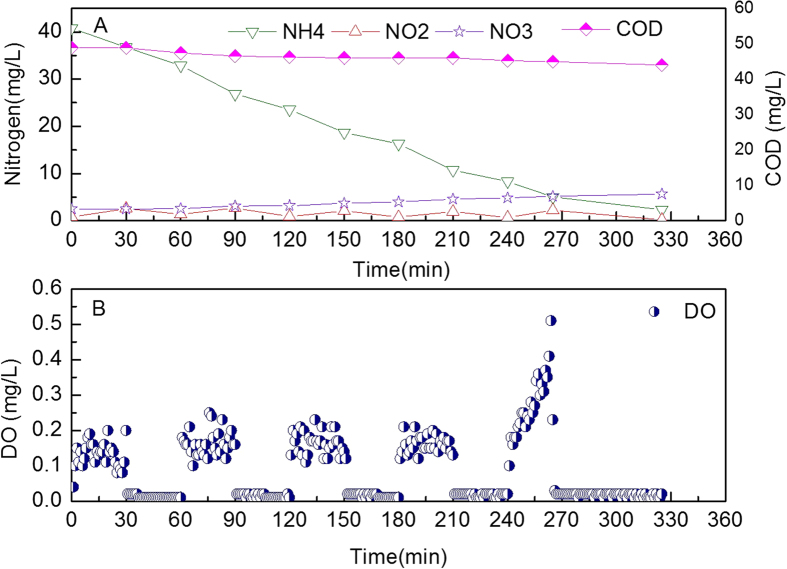
Nitrogen concentrations (**A**) and dissolved oxygen (DO) concentrations (**B**) in a typical cycle.

Detailed investigations of the nitrogen conversion in the reaction were performed through cycle studies, with an example presented in Fig. 4 (day 30). During the aeration stage, the DO concentration was 0.08–0.25 mg/L (Fig. 4), and the average DO was 0.15 mg/L. Under aerobic conditions, ammonium was oxidized and nitrite accumulated. At the end of the first aeration stage, the nitrite concentration was 2.54 mg/L (Fig. 4). In the subsequent anoxic stage, the nitrite concentration decreased from 2.54 mg/L to 1.37 mg/L, and the ammonium concentration decreased from 36.7 mg/L to 32.9 mg/L. In the next aerobic and anoxic sub-cycle, the same phenomenon occurred. In the fifth aeration stage, DO began to increase rapidly. The ammonium concentration dropped to 5.0 mg/L when DO increased to 0.5 mg/L. Then aeration was stopped, and a 60-minute anoxic stage began. At the end of this cycle, the ammonium, nitrite and TN concentrations were 2.3 mg/L, 0.2 mg/L and 8.4 mg/L, respectively.

### Nitrogen removal performance of the nitritation/anammox reactor treating domestic wastewater using intermittent aeration with low DO

**Figure 5:**
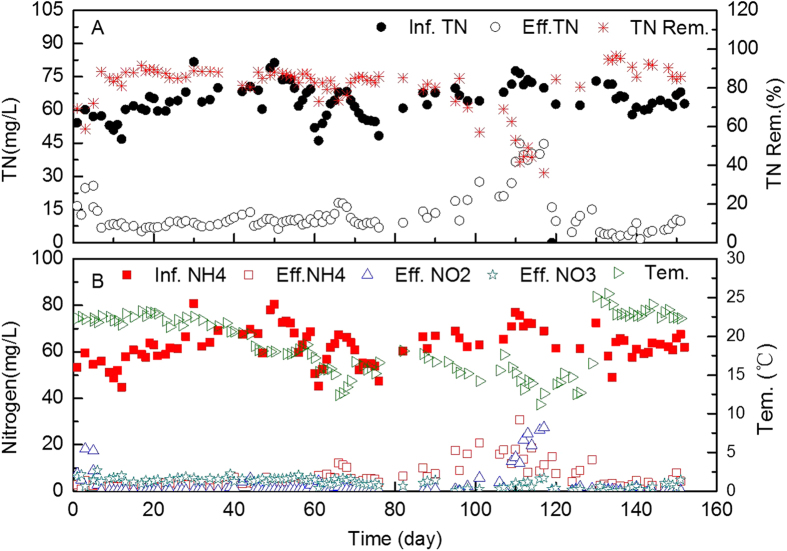
Total nitrogen (TN) concentrations in the influent (Inf.TN) and effluent (Eff.TN), and TN removal efficiencies (Re. TN); NH_4_^+^-N concentrations in the influent (Inf.NH4) and NH_4_^+^-N (Eff.NH4), NO_2_^−^-N (Eff.NO2) and NO_3_^−^-N (Eff.NO3) concentrations in the effluent.

Nitrogen was efficiently removed from domestic wastewater with anammox above 20 °C. After start-up, the TN removal rate was 87%, on average (Fig. 5). The average effluent TN concentration was 8.6 mg/L with an average influent TN of 62.2 mg/L. The effluent nitrite concentration was maintained below 0.6 mg/L. When temperature decreased from 20.0 °C to 18.3 °C during the last eight days of phase I (day 36 to 44), the effluent nitrite concentration increased from 0.3 to 5.5 mg/L. Then, anoxic time was extended from 30 to 60 minutes on day 45, to enhance the anammox reaction and avoid nitrite accumulation. After that modification, the effluent nitrite and TN concentrations dropped to 0.26 mg/L and 7.7 mg/L at once. However, the effluent nitrite once again climbed to 4.3 mg/L when the temperature dropped to 15.3 °C at the end of phase II. Anoxic time was again extended in phase III, and the aerobic time was decreased to 15 minutes. However, the effluent nitrite and TN concentrations still went up to 27.3 mg/L and 44.7 mg/L when the temperature fell to 10.5 °C on day 117.

Then, the mixer with two impellers was changed to one mixer with one impeller, to decrease the oxygen transfer from air to liquid during the anoxic stage. The average effluent nitrite concentration decreased to 0.7 mg/L, and the average effluent TN decreased to 9.4 mg/L between day 119 and 132 (Fig. 5). In phase IV, a heater was installed in the reactor, increasing the temperature increase to 21.6 °C. Anammox activity increased with the temperature increase, and the anoxic time was decreased from 90 to 15 minutes. After heating, good nitrogen removal performance was achieved between day 139 and 151. The average effluent nitrite and TN were 0.5 mg/L and 6.3 mg/L, respectively; and TN could be removed from domestic wastewater with an average removal rate of 88.3%. This result indicates that it is still a challenge to achieve good nitrogen removal form domestic wastewater with anammox at low temperatures (e.g. below 15 °C).

## Discussion

### The role of intermittent aeration with low DO on suppressing NOB growth in the nitritation/anammox reactor

Suppressing NOB growth is a necessary prerequisite for removing nitrogen from domestic wastewater with anammox^10^,^11^,^18^. Many factors have been identified in the literature that selectively inhibit or limit NOB growth, leading to nitritation/anammox or nitritation/denitrification. These factors include high temperature^19^, high ammonium/ammonia concentration^20^,^21^, high nitrite/free nitrous acid concentration^22^,^23^, high salt concentration^24^, and low DO concentration^25^,^26^. The effect of temperature on suppressing NOB growth has been demonstrated and widely proposed as the main mechanism for achieving the nitrite pathway in the SHARON process^19^. However, it is believed that temperature is generally more a disturbance than a real control parameter in most full-scale processes. It is difficult to have strong FA and FNA inhibition of the NOB population without having high levels of ammonium and nitrite in the influent or effluent of the domestic wastewater treatment system^27^,^28^. While high salt concentration has been demonstrated to cause higher reduction in NOB growth than in AOB growth^24^, this applies only for specific types of domestic wastewater, and is therefore not generally available for achieving nitritation in domestic wastewater treatment system. Although changing domestic wastewater characteristics to achieve nitritation is difficult, it may be possible to optimize the operational conditions to suppress NOB growth. Intermittent aeration with low DO is proposed to control NOB growth through optimized conditions.

FISH probes were applied for *Nitrobacter* and *Nitrospira*, which are typically the dominating NOB in wastewater treatment plants. The results clearly showed that the sizes for both populations were maintained at low levels of 2.0–2.6% (Fig. 3), and hence it is convincing to conclude that the growth of both of these known, and typically dominating, NOB was suppressed. The ratio of nitrate production to ammonium oxidation was 6.2 ± 2.8%, this value was lower than 11%, the theoretical nitrate production ratio for the nitritation and anammox process^29^. This is likely because that some of the nitrate produced was removed by denitrification, as the wastewater does contain a low level of COD. The low level of nitrate accumulation also strongly suggests the low ratio of nitrate production, along with the high nitrogen removal efficiency, strongly suggests that nitrate production by NOB would be limited, and the nitritation and anammox process is primarily responsible for the high-level of nitrogen removal in the system. These results corroborate the NOB abundance data, indicating that NOB was suppressed in the reactor.

NOB mainly grew in the aerobic stage of the intermittent aeration reactor. The substrates of NOB, oxygen, and nitrite produced by AOB coexisted during the aerobic stage. However, nitrite, the energy source for NOB, accumulated at the end of aerobic stage (Fig. 4). This means that NOB growth is lower than AOB growth. In the subsequent anoxic stage, NOB could not grow because it lacked oxygen. After many cycles of operation, AOB outnumbers NOB. Peng *et al.*^30^ used this method to achieve nitritation in a SBR treating domestic wastewater. In that experiment, the ammonia depletion point was detected using DO as a parameter. Aeration was then terminated immediately to accumulate nitrite and to avoid continuous nitrite oxidation. Yang *et al.*^27^ achieved nitrite accumulation to remove nitrogen from municipal wastewater with nitritation/denitritation in a step-feed sequencing batch reactor (SBR) under alternating aerobic/anoxic operational conditions. Low DO also benefited the suppression of NOB growth since the NOB growth rate is lower than AOB under low DO concentration^10^,^13^,^25^. This indicated that intermittent aeration with low DO resulted in less NOB growth than AOB.

TN removal was observed in the aeration stage of the current study. This TN loss was mainly due to the anammox reaction since denitrification is very limited because of limited organic matter. So, anammox bacteria consumed nitrite in the aeration stage. Meanwhile, anammox bacteria also reduced nitrite in the anoxic stage (Fig. 4). However, NOB can only oxidize nitrite in the aeration stage, because NOB can not obtain oxygen as a substrate in the anoxic stage. Therefore, anammox bacteria have an obvious advantage in the competition of nitrite with NOB in the intermittent aeration nitritation/anammox reactor. The low DO also makes anammox bacteria a lot more competitive, as a decrease in DO concentration results in a decrease of NOB activity, and reduces its potential inhibition on anammox bacteria^31^. From the above discussion, it can be concluded that suppressing NOB growth is feasible using intermittent aeration coupled with low DO.

### Achieving nitrogen removal from domestic wastewater via anammox using intermittent aeration with low DO

Suppressing NOB growth was achieved in this study by using intermittent aeration. At the same time, it was found that anammox was primarily contained in granular sludge, and the anammox bacteria abundance held at between 1.89 ± 0.62 × 10^10^ copies/g MLSS and 2.33 ± 0.08 × 10^10^ copies/g MLSS over the entire experimental period. This means that a good retention of anammox bacteria could be achieved through the formation of granular sludge, which was also found in previous studies^8^,^12^,^32^. This microbiological structure provides grounds for achieving nitrogen removal from wastewater with anammox.

Nitrogen removal performance showed that nitrogen could be efficiently removed from domestic wastewater above 20 °C. Nitrite began accumulating in the reactor when the temperature fell below 20 °C; nitrite concentration went up to 27.3 mg/L at 10.5 °C. This was due to the decrease in anammox bacteria activity. Nitrite accumulation was also observed in other studies when temperature decreased to 15 °C^32^,^33^, 13 °C^34^, 10 °C^35^ and 9 °C^9^. When the temperature was subsequently increased, nitrite accumulation disappeared, due to the recovery of anammox bacteria activity. These results show it remains a challenge to achieve good nitrogen removal from domestic wastewater with anammox at low temperatures. Controlling DO concentration is a method to limit AOB activity, further reducing nitrite production and avoiding nitrite accumulation^32^. In the current study, extending anoxic time was proven to be an effective way to reduce nitrite accumulation.

This study investigated the feasibility of using intermittent aeration with low DO to suppress NOB growth, with the goal of removing nitrogen from domestic wastewater with anammox. The main conclusions were: (1) NOB growth was successfully suppressed by applying intermittent aeration with low DO. (2) Good retention of anammox bacteria was achieved because it mainly accumulated in granular sludge. (3) Nitrogen was effectively removed from domestic wastewater via anammox above 20 °C. Effluent TN was 6.64 mg/L when influent TN and COD_soluble_ were 62.59 mg/L and 87.95 mg/L, respectively. However, low temperatures create a challenge for nitrogen removal.

## Methods

A one-stage nitritation/anammox reactor was built to treat real domestic wastewater, which had been pretreated in a high rate activated sludge reactor. The characteristics of the feeding domestic wastewater to the one-stage nitritation/anammox reactor were as follows: soluble chemical oxygen demand (COD_soluble_) = 76.6 ± 30.7 mg/L, NH_4_^+^-N = 62.6 ± 7.6 mg/L, NO_2_^−^-N = 1.1 ± 2.7 mg/L and NO_3_^−^-N = 0.2 ± 0.3 mg/L.

The seeding sludge was obtained from a pilot-scale nitrite/anammox reactor treating N-rich wastewater under 27–29 °C, which was located in the Beijing Gaobeidian WWTP. This pilot-scale reactor was an integrated fixed-biofilm activated sludge (IFAS) reactor, with a working volume of 12 m^3^ separated into five equal sections. The first section was set as the anoxic phase, and was continually stirred by a mechanical mixer. The other zones were aerated by a compressor through fine diffusers installed at the bottom of the bioreactor. The carrier material, cubic shaped sponges comprised of polyester (BioCube Co., Ltd., Korea), was fixed in the aerobic zones, occupying 15% of the aerobic zone volume. Small granular sludge was present in the activated sludge, which was attributed to the addition of powdered activated carbon. The nitrogen loading rate of this reactor was approximately 0.6 kg N/m^3^/d. The nitrogen removal rate was 60–70%.

A lab-scale SBR made of plexiglass with a working volume of 10L was used in this study. A pH, DO and temperature online sensor (WTW 340i, Germany) were installed. Air was varied intermittently to create aerobic and anoxic conditions, achieved by using a PLC control system to turn on and turn off the air compressor. A mechanical stirrer was used to provide liquid mixing under anoxic phase. During the aeration stage, an air-compressor was used to supply oxygen. Each cycle of the SBR system consisted of filling, alternate aeration and anoxic mixing, settling and decanting. The alternate aeration and anoxic mixing resulted in many repeated cycles of aerobic time (T_1_) and anoxic time (T_2_). The air flow rate was kept constant and the mean DO was approximately 0.15 mg/L during aerobic ammonium oxidation. After ammonium oxidation was complete, DO began increasing. Once DO increased to 0.5 mg/L, the air compressor was shut down, and the anoxic period of the last anoxic phase in an overall SBR cycle (T_3_) started. T_1_, T_2_ and T_3_ are shown in Table 1. The settling time was 30 minutes, and decanting lasted 10 minutes. One cycle was operated in one day. The operation plan and conditions of nitritation/anammox SBR are shown in the Table 1. During phase I–III the reactor temperature was not controlled, and a heater was used to heat the reactor during the phase IV.

Soluble chemical oxygen demand (COD_soluble_) was measured according to standard methods (APHA 1995). To control the nitrite interference with COD measurement, the COD measured was corrected by subtracting the contribution of nitrite on the basis of 1.1 g COD/g NO_2_^−^-N. Ammonium concentrations (NH_4_^+^-N), nitrite concentrations (NO_2_^−^-N), and nitrate concentrations (NO_3_^−^-N) were all measured using Quickchem^®^ 8500 (Hach Company, Lachat Instruments). All samples were filtered through a 0. 5 um filter before analyzing. DO, pH, and temperature were measured by oxygen, pH and temperature probes (WTW 340i, WTW Company). The particle size distribution of the granular sludge was analyzed by using a Malvern Mastersizer 2000 particle size analyzer. The activated sludge was divided into two groups with different particle sizes using the wet-sieving method. Compared to dry sieving, wet-sieving could obtain a better separation between the individual fractions. The wet-sieving process was supported by water from a spray nozzle, which was located above the sieve. The samples were rinsed until the liquid leaving the sieve was no longer turbid.

DNA was extracted from 0.10 g of dry activated sludge using the Fast DNA SPIN Kit for Soil (QBIOgene Inc., Carlsbad, CA, USA), with a beating time of 45 s and a speed setting of 5.5 m. DNA was stored at −20 °C for further analyses. DNA concentration was determined on a Nanodrops ND-1000 UV-Vis Spectrophotometer (NanoDrop Technologies, Wilmington, DE, USA).

The abundance of anammox bacteria and AOB were determined using a Stratagene Mx3005p QPCR system (Agilent Technologies, USA) with the SYBR-Green approach (TAKARA, Dalian, China). The hydrazine synthase (hzs) function gene of anammox and amoA function gene of AOB were amplified using the HSBeta396F/HSBeta742R and amoA-1f/amoA-2r, respectively. Positive clones of hzsB and amoA were selected to isolate plasmid DNA using a GeneJet Plasmid Miniprep Kit (Fermentas MBI, Lithuania) as the gene standard. The concentration of plasmid DNA was determined on a Nanodrops ND-1000 UV-Vis Spectrophotometer for calculating target gene copy numbers. Standard curves were obtained with tenfold serial dilutions of the plasmid DNAs. The results with efficiency and correlation coefficient above 95% and 0.98 were used. Each quantitative PCR reaction was performed in triplicate. For anammox gene amplification, 20 μl reaction mixtures, including 10 μl SYBR^®^ Premix Ex Taq™ (Takara, Dalian, China), 0.4 μl ROX Reference Dye50, 0.21 μl each primer (10 μM) and 2 μl DNA template (1–10 ng) were used. The anammox gene amplification program consisted of the following steps: 3 min at 95 °C, followed by 40 cycles of 30 s at 95 °C, 30 s at 59 °C, and 30 s at 72 °C. For AOB gene amplification, 20 μl reaction mixtures were used, which include 10 μl SYBR^®^ Premix Ex Taq™ (Takara, Dalian, China), 0.4 μl ROX Reference Dye50, 0.2 μl each primer (10 μM) and 2 μl DNA template (1–10 ng). The AOB gene amplification program consisted of the following steps: 3 minutes at 95 °C, followed by 40 cycles of 30 s at 95 °C, 30 s at 55 °C, and 30 s at 72 °C.

FISH probes were applied for *Nitrobacter* and *Nitrospira*, which are typically the dominating NOB in wastewater treatment plants. Sample fixation and hybridization steps were carried out according to methods previously described by Amann, *et al.*^36^. FISH was performed with EUBmix (EUB338, EUB338-II, EUB338-III) specific for members of the domain bacteria, NIT3 specific for *Nitrobacter* and Nstpa662 specific for *Nitrospira*^37^. The images of FISH samples were captured using an OLYMPUS-BX52 fluorescence microscope. FISH quantification was carried out using Image-pro plus 6.0 Software^®^, where the relative abundance of the interested bacteria was determined as the mean percentage of all bacteria.

## Additional Information

**How to cite this article**: Ma, B. *et al.* Suppressing Nitrite-oxidizing Bacteria Growth to Achieve Nitrogen Removal from Domestic Wastewater via Anammox Using Intermittent Aeration with Low Dissolved Oxygen. *Sci. Rep.* **5**, 13048; doi: 10.1038/srep13048 (2015).

## Supplementary Material

Supplementary Information

## Figures and Tables

**Figure 1:**
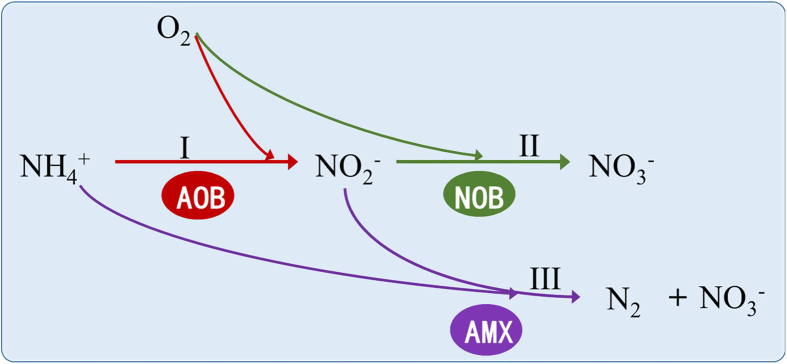
A schematic of biological nitrogen conversion pathway in WWTPs, AOB: ammonium oxidation bacteria, NOB: nitrite oxidation bacteria, AMX: anaerobic ammonium oxidation bacteria.

**Table 1:** Nitritation/anammox sequencing batch reactor (SBR) operation schedule.

Phase	Day	Aerobic(T_1_)/Anoxic(T_2_) (min)	Lastanoxic(T_3_)(min)	Tempreture(°C)
Phase I	1–44	30/30	60	18–22
Phase II	45–61	30/60	60	15–18
Phase III	62–130	15/90	90	11–17
Phase IV	131–152	15/15	30	19–23
