# Supplementary material for: Suppressing Nitrite-oxidizing Bacteria Growth to Achieve Nitrogen Removal from Domestic Wastewater via Anammox Using Intermittent Aeration with Low Dissolved Oxygen
Source: Sci Rep. 2015 Sep 10;5:13048. doi: 10.1038/srep13048 (PMC4564738; doi:10.1038/srep13048)
Supplement: Supplementary Information [file srep13048-s1.doc]

**Supplementary information**

**Suppressing Nitrite-oxidizing Bacteria Growth to Achieve Nitrogen Removal from Domestic Wastewater via Anammox Using Intermittent Aeration with Low Dissolved Oxygen**

Bin Ma^1^, Peng Bao^1^, Yan Wei^1^, Guibing Zhu^2^, Zhiguo Yuan^1,3^, Yongzhen Peng^1, *^

1. Key Laboratory of Beijing Water Quality Science and Water Environment Recovery Engineering, Beijing University of Technology，Beijing, China;

2.State Key Laboratory of Environmental Aquatic Quality, Research Center for Eco-Environmental Sciences, Chinese Academy of Sciences, Beijing, China;

3. Advanced Water Management Center, The University of Queensland, St Lucia, QLD 4072, Australia.

Correspondence and requests for materials should be addressed to Y. P. (Yongzhen Peng).

E-mail: (pyz@bjut.edu.cn)


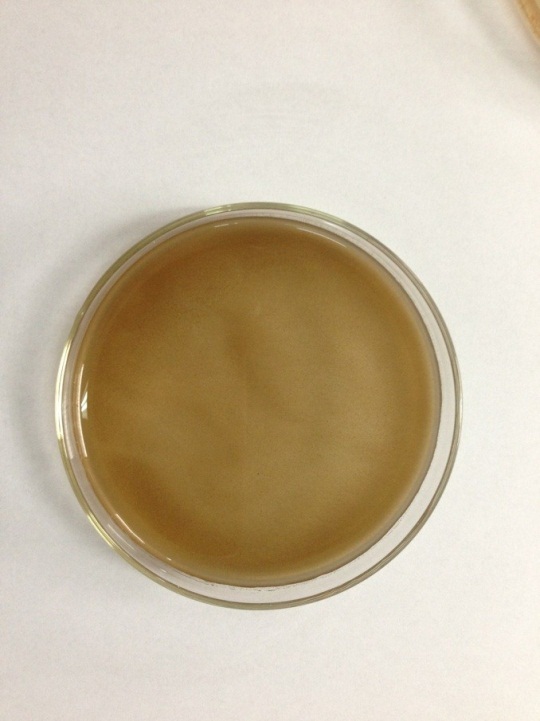

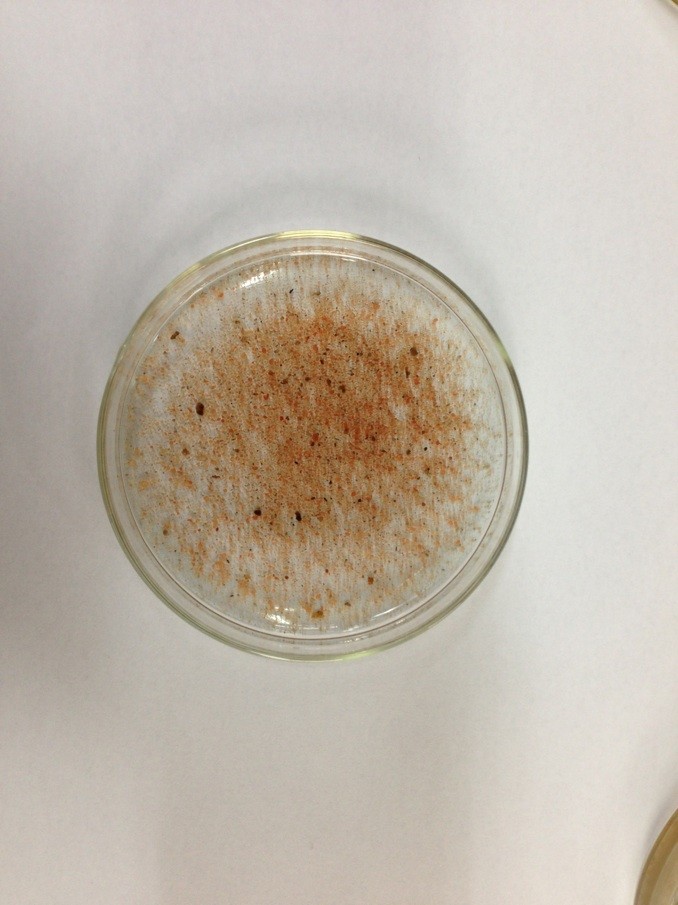

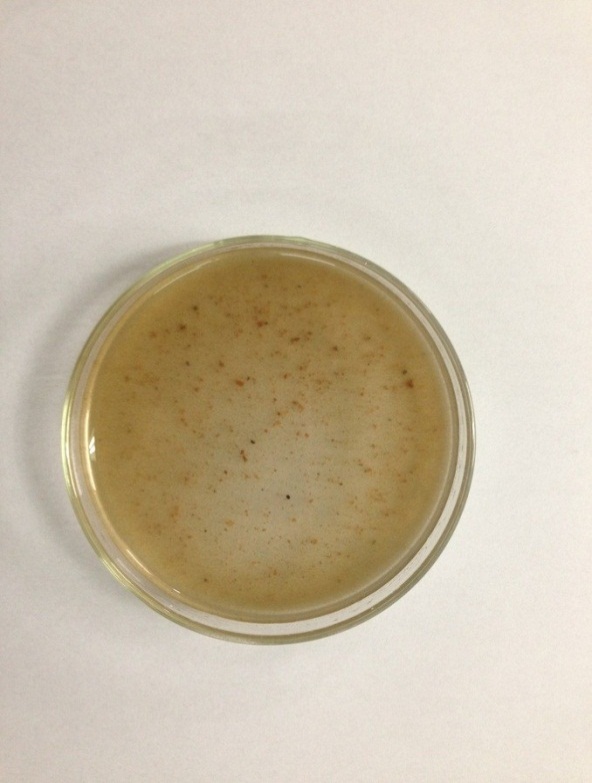


Granular sludge

Activated sludge

Activated sludge flocs

**Figure S1 the photo of mixed activated sludge, granular sludge ( > 200 μm) and activated sludge flocs ( < 200 μm).**

| **Table S1 Overview of particle size, settling velocity, nitrogen removal rate of anammox granular sludge** | | | |
| --- | --- | --- | --- |
| Particle size  (mm) | NRR  (kg N/m^3^/d) | settling velocity  (m/h) | Source cited |
| 2.5 | 0.86 | 41-79 | 1 |
| 1.0 | 6.39 | 21-54 | 2 |
| 0.8 | 5.7 | 25-51 | 3 |

References

1 Ni, B. J., et al. Microbial and Physicochemical Characteristics of Compact Anaerobic Ammonium-Oxidizing Granules in an Upflow Anaerobic Sludge Blanket Reactor. *Appl. Environ. Microb.* **76**, 2652-2656, (2010).

2 Imajo, U., Tokutomi, T. & Furukawa, K. Granulation of anammox microorganisms in up-flow reactors. *Water Sci. Technol.* **49**, 155-163, (2004).

3 Ma, B. et al. Performance of anammox reactor treating low strength wastewater under moderate and low temperature, *Bioresour. Technol.* **129**, 606-611, (2013).
